# Prognostic Roles of Inflammatory Markers in Pancreatic Cancer: Comparison between the Neutrophil-to-Lymphocyte Ratio and Platelet-to-Lymphocyte Ratio

**DOI:** 10.1155/2018/9745601

**Published:** 2018-06-07

**Authors:** Dongwook Oh, Jung-Soo Pyo, Byoung Kwan Son

**Affiliations:** ^1^Department of Gastroenterology, Asan Medical Center, University of Ulsan College of Medicine, Seoul, Republic of Korea; ^2^Department of Pathology, Eulji University Hospital, Eulji University School of Medicine, Daejeon 34824, Republic of Korea; ^3^Department of Internal Medicine, Eulji Hospital, Eulji University School of Medicine, Seoul 01830, Republic of Korea

## Abstract

**Background/Objectives:**

This meta-analysis is aimed at investigating the prognostic roles of the inflammatory markers neutrophil-to-lymphocyte ratio (NLR) and platelet-to-lymphocyte ratio (PLR) in patients with pancreatic cancer.

**Methods:**

The correlations between high inflammatory marker expression levels and prognosis in 7105 patients with pancreatic cancer from 34 eligible studies were investigated. Additionally, subgroup analyses based on study location, tumor stage, treatment, and value cutoffs were performed.

**Results:**

High NLR and PLR values were considered to be 2.0–5.0 and 150–200, respectively. Using a random-effects model, the estimated rates of high NLR and PLR were 0.379 (95% confidence interval [CI] 0.310–0.454) and 0.490 (95% CI 0.438–0.543), respectively. High NLRs were frequently found in patients with lower tumor stages and in those who underwent surgery. There were significant correlations between high NLR and PLR and poor survival rates (hazard ratio [HR] 1.737, 95% CI 1.502–2.009 and HR 1.143, 95% CI 1.037–1.259, resp.). Interestingly, the NLR and PLR had no prognostic value in patients who underwent chemoradiotherapy.

**Conclusion:**

Taken together, our results showed that inflammatory markers are useful for predicting prognosis in patients with pancreatic cancer. The NLR is a more suitable parameter for predicting prognosis regardless of the patient's condition.

## 1. Introduction

Pancreatic cancer (PC) is one of the most lethal malignant neoplasms in the world [1]. The long-term prognosis of patients with PC is poor, with a median survival of 8.5–11 months for those with metastatic disease even with aggressive treatment [2, 3]. To date, surgical excision is the only curative treatment for PC; however, only 10–15% of patients are eligible for this procedure [4]. Chemo/radiotherapy is used to palliate symptoms and improve survival in patients with advanced disease. Therefore, accurate clinical staging and identification of prognostic factors are crucial for estimating prognosis and selecting appropriate treatment modalities.

Although the pathological stage of PC is considered the most significant prognostic factor, it is difficult to obtain tumor tissues for analysis in a significant number of patients. Recently, several studies have demonstrated that the neutrophil-to-lymphocyte ratio (NLR) and platelet-to-lymphocyte ratio (PLR) could serve as a simple immune function index and may be of prognostic significance in patients with various solid tumors [5, 6]. However, the relationship between NLR/PLR and clinical outcomes in patients with PC remains controversial. As they are derived from routine laboratory tests, NLR and PLR are easy to obtain and may serve an important function in monitoring PC progression as well as in predicting patient survival. Therefore, we performed this study to evaluate the prognostic values of NLR and PLR in patients with PC.

## 2. Methods

### 2.1. Published Studies' Search and Selection Criteria

We followed the methods of Jeong et al. [7]. Relevant articles were obtained by searching the PubMed and MEDLINE databases through December 31, 2017. These databases were searched using the following key words: “pancreatic cancer,” “survival,” and “neutrophil-to-lymphocyte ratio or platelet-to-lymphocyte ratio.” The titles and abstracts of all searched articles were screened. Review articles were also screened to identify additional eligible studies. Articles related to studies of human PC (other than pancreatic neuroendocrine tumors) and those with data pertaining to the correlation between inflammatory markers and survival were included. Articles were excluded if they were case reports or nonoriginal articles, or if not written in English. This protocol was reviewed and approved by the Institutional Review Board of Eulji Hospital (approval number NON2017-002).

### 2.2. Data Extraction

Data from all eligible studies were extracted by two independent authors. The following data were extracted from each of the eligible studies [8–41]: the first author's name, year of publication, study location, number of patients analyzed, tumor stage, treatment modality, criteria for each inflammatory marker, rate of patients with high inflammatory marker values, and information on the correlations between inflammatory markers and survival. For quantitative aggregation of survival results, the correlations between inflammatory markers and the overall survival (OS) rates were analyzed according to the reported hazard ratio (HR) using one of three methods. In studies not quoting the HR or its confidence interval (CI), these variables were calculated from the presented data using the HR point estimate, log-rank statistic or its *P* value, and the *O*-*E* statistic (i.e., the difference between the numbers of observed and expected events) or its variance. If these data were unavailable, the HR was estimated using the total number of events, number of patients-at-risk in each group, and the log-rank statistic or its *P* value. Finally, if the only useful data were in the form of graphical representations of survival distributions, survival rates were extracted at specified times to reconstruct the HR estimate and its variance under the assumption that patients were censored at a constant rate during the time intervals [42]. The published survival curves were read independently by two authors to reduce variability. The HRs were then combined into an overall value using Peto's method [43].

### 2.3. Statistical Analyses

To perform the meta-analysis, all data were analyzed using the Comprehensive Meta-Analysis software package (Biostat, Englewood, NJ, USA). We investigated high values of inflammatory markers (the NLR and PLR) and their correlations with OS rates in patients with PC. Heterogeneity between the studies was checked by the *Q* and *I*^2^ statistics and expressed as *P* values. Additionally, sensitivity analysis was conducted to assess the heterogeneity of eligible studies and the impact of each study on the combined effect. Because eligible studies were evaluated in various populations with different tumor stages and treatments, a random-effects model was applied as it was more suitable than a fixed-effects model for interpreting the influence of inflammatory markers. Begg's funnel plot and Egger's test were used to assess publication bias; if significant publication bias was found, fail-safe *N* and trim-fill tests were performed to determine the degree of such bias. The results were considered statistically significant at *P* < 0.05.

## 3. Results

### 3.1. Selection and Characteristics of the Studies

**Figure 1:**
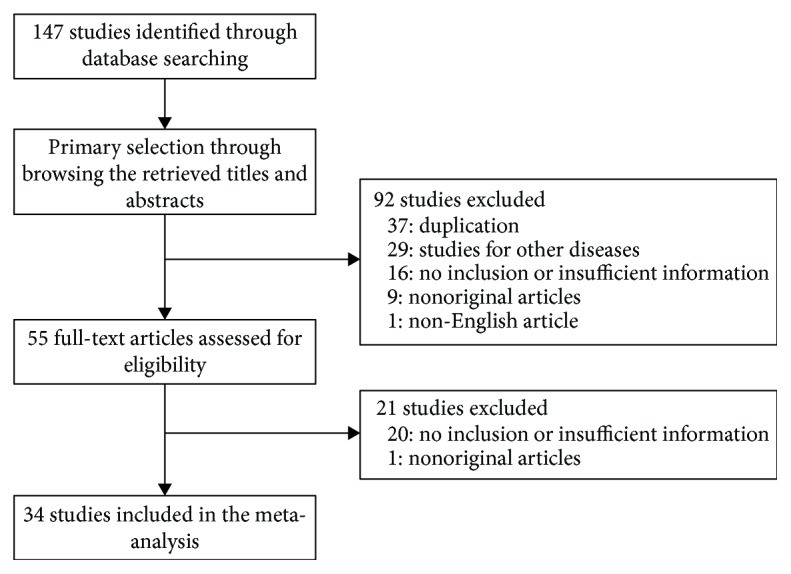
Flow diagram of the study selection process.

One hundred forty-seven reports were retrieved from the database; 37 articles were excluded as they were duplicates while a further 36 were excluded because of insufficient or no information. Additionally, 40 reports were excluded because they described other diseases (*n* = 29), were published in a language other than English (*n* = 1), or were nonoriginal research articles (*n* = 10). Finally, 34 studies that encompassed 7105 patients with PC were included in this meta-analysis (Figure 1 and Table 1).

### 3.2. Meta-Analysis

First, the relationships between high NLR and PLR values and PC were investigated. Overall, the rates of high NLR and PLR as determined using the random-effects model were 0.379 (95% CI 0.310–0.454) and 0.490 (95% CI 0.438–0.543), respectively. Additional data, as well as the results of heterogeneity and publication bias analyses, are shown in Table 2. Patients with lower tumor stages (I and II) showed higher NLRs than those with higher tumor stages (III and IV). Additionally, the rate of high NLR was higher in patients who had undergone surgery than in those who received other treatments. There were no differences in the rates of high NLR and PLR between study locations. In comparison between higher and lower criterion subgroups, higher criteria of NLR showed significantly higher rate of high NLR than lower criteria of NLR. However, in PLR, there was no significant difference between higher and lower criteria of PLR.

Next, the correlations between high values of these inflammatory markers and OS rates were investigated. High NLR and PLR values were significantly correlated with poorer OS (HR 1.737, 95% CI 1.502–2.009 and HR 1.143, 95% CI 1.037–1.259, resp.; Table 3). Subgroup analyses based on study location, tumor stage, treatment, and cutoffs were conducted. With respect to NLR, all subgroups except for patients who underwent chemotherapy and/or radiotherapy showed significant correlations between high NLR and a poorer OS; a high NLR had no prognostic role in patients who underwent chemo- or radiotherapy (HR 0.922, 95% CI 0.269–3.162). Patients with high PLR values only showed poorer OS if they were Asian or underwent mixed treatment (i.e., surgery plus chemo/radiotherapy).

## 4. Discussion

In this meta-analysis of 34 studies comprising 7105 patients with PC, we showed that the NLR and PLR constitute novel prognostic markers for predicting the prognosis of patients with PC. To the best of our knowledge, our meta-analysis is the first to investigate this relationship. The results of our meta-analysis demonstrated that high NLR and PLR values were found to be correlated with poor OS in patients with PC.

Although there was no significant difference in PLR among patients in the present series, a high NLR was frequently found in patients with lower tumor stages (I and II) and in those who had undergone surgery. In view of the relatively high likelihood of poor outcomes in patients with PC regardless of stage, patients with lower-stage tumors that are usually limited to the pancreas are most likely to undergo surgical resection [44]. The prognosis is better in patients with lower tumor stage than in those with higher stage [45]. In other types of cancer, a lower tumor stage was associated with lower NLR levels [46, 47]. In our study, the subgroup with low tumor stages (I-II) showed higher rate of high NLR than the subgroup with high tumor stages (III-IV) (0.656 versus 0.373). However, because the subgroup with low tumor stage included only one eligible study, further studies will be needed to obtain the detailed information of PC with low stage. A plausible explanation for our apparently counterintuitive results is that a subset of patients included in the analysis underwent palliative surgery [9, 35–37]. Moreover, the time of obtaining blood samples for measuring neutrophils, lymphocytes, and platelets before treatment might be an important limiting factor. Another possible bias in our data is the presence of biliary sepsis; approximately 56% of PC patients present with obstructive jaundice and are more susceptible to bacterial infection owing to bile duct obstruction [48, 49]. Few studies included in our meta-analysis controlled for biliary infection by excluding patients with this septic condition; therefore, such comorbidities may have influenced our findings [13, 26, 28].

The exact mechanisms between high NLR/PLR values and poor outcomes in patients with PC are unclear. Systemic inflammation plays decisive roles at different stages of tumor development, including initiation, promotion, malignant conversion, invasion, and metastasis. Inflammation may enhance tumor initiation through genetic mutations, genomic instability, and epigenetic modifications and can activate tissue repair responses that induce proliferation of premalignant cells and prolong their survival. Inflammation also stimulates angiogenesis, causes immunosuppression, and promotes the formation of tumor-supporting microenvironments that ultimately promote metastasis [50]. The close association between increased systemic inflammatory responses (as assessed by NLR and PLR) and poor prognosis may also be related to cancer cell activation of inflammatory processes. Cancer-related inflammation suppresses antitumor immunity by recruiting regulatory T cells and activating chemokines, resulting in tumor progression. Tumors also secrete vascular endothelial growth factor (VEGF), a vascular permeability factor that induces persistent extravasation of fibrin and fibronectin and continuous generation of the extracellular matrix [51]. Platelets are a critical source of cytokines, especially transforming growth factor-beta as well as VEGF, which can promote cancer progression by enhancing angiogenesis [50–52]. Proinflammatory cytokines such as interleukins 1 and 6 can promote megakaryocyte proliferation; this results in thrombocytosis, which is a negative prognostic marker in several cancers [53–55]. Therefore, inflammatory markers might be an indicator of prognosis. Recently, NLR and PLR have now been investigated as prognostic factors. The measurement of the NLR and PLR is straightforward and convenient and is potentially useful in daily oncologic practice.

Our study found that patients with PC who have high NLR values exhibit poor OS which was consistent with the results in other types of malignancies [46, 47, 56]. Subgroup analysis of NLR stratified by study location, tumor stage, treatment, and threshold criteria also demonstrated that a high NLR had a negative effect on OS except in patients who underwent chemotherapy and/or radiotherapy. The NLR after chemo- and radiotherapy did not correlate with OS. NLR is a relative value that fluctuates depending on neutrophil or lymphocyte changes and may therefore be affected by chemotherapy, radiotherapy, or granulocyte colony-stimulating factor administration [29]. These factors might induce changes in the number of neutrophils or lymphocytes; clinicians should consider these conditions in clinical practice. Several studies demonstrated that postchemotherapy NLR change was an independent prognostic marker [8, 29, 32]. Given potential chemotherapy- or radiotherapy-related toxicities, increased NLR values after treatment may help physicians decide to transfer affected patients to early palliative care, whereas a decrease in the NLR after chemo- or radiotherapy can be considered an early predictor of response to treatment.

High PLR could also predict OS of patients with PC in accordance with other malignancies [46, 47, 56]. However, in subgroup analyses, a high PLR was associated with worse OS only in Asian patients and in those who underwent mixed treatment (surgery plus chemo/radiotherapy). Our meta-analysis demonstrated that NLR is a better predictor of prognosis of patients with PC than PLR, which is also consistent with the results of previous studies [35, 57]. Our analysis may provide important information to support treatment decision-making, including pursuing more aggressive treatments.

There were several limitations in this meta-analysis. First, all of the included studies were retrospective and were thereby more prone to some biases. Second, information about PLR in patients who underwent surgical treatment could not be obtained from the eligible studies. Third, a comparison between pre- and posttreatment inflammatory marker values could not be performed owing to insufficient information.

In conclusion, high NLR and PLR values are useful predictors of worse survival in patients with PC. These parameters can therefore be useful for identifying high-risk patients with PC and for determining individual treatment plans.

## Figures and Tables

**Table 1:** Main characteristics of the studies included in this meta-analysis.

Author, year	Location	Tumor type	Tumor stage	Tx option	Parameter	Criteria	Criterion subgroup	Number of patients		
								Total	High	Low
Alagappan et al., 2016 [24]	USA	PDAC	ND	Mixed	NLR	5	High	208	ND	ND
					PLR	200	High	208	ND	ND
An et al., 2010 [36]	China	PC	III-IV	CTx	NLR	5	High	95	ND	ND
Asaoka et al., 2016 [21]	Japan	PC	I-III	Mixed	NLR	2.7	Low	46	20	26
Asari et al., 2016 [22]	Japan	PDAC	I-IV	Mixed	NLR	3	Low	184	62	122
Ben et al., 2015 [28]	China	PDAC	I-III	Surgery	NLR	2	Low	381	267	114
Chawla et al., 2017 [37]	USA	PDAC	I-IV	Mixed	NLR	3.3	Low	217	107	110
					PLR	175	High	217	107	110
Chen et al., 2017 [10]	China	PDAC	III-IV	CTx	NLR	2.78	Low	132	78	54
Cheng et al., 2016 [25]	China	PC	I-II	Mixed	NLR	2	Low	195	128	67
Inoue et al., 2015 [30]	Japan	PDAC	I-IV	Mixed	NLR	2	Low	440	300	140
					PLR	150	Low	440	201	239
Kishi et al., 2015 [27]	Japan	PC	III/IV	CTx/RTx	NLR	5	High	65	7	58
					PLR	150	Low	65	30	35
Kou et al., 2016 [23]	Japan	PC	III-IV	CTx	NLR	5	High	306	49	257
					PLR	150	Low	306	180	126
Lee et al., 2016 [19]	Korea	PDAC	III-IV	CTx	NLR	5	High	82	20	62
					PLR	150	Low	82	36	46
Liu et al., 2017 [14]	China	PDAC	I-IV	Mixed	NLR	4.5	High	386	63	323
Luo et al., 2015 [29]	China	PDAC	III-IV	CTx	NLR	3.1	Low	403	194	209
Martin et al., 2014 [31]	Australia	PC	III-IV	CTx/RTx	NLR	5	High	124	60	64
					PLR	200	Low	124	75	49
Mitsunaga et al., 2016 [41]	Japan	PC	III-IV	CTx	NLR	5	High	195	ND	ND
Montes et al., 2017 [11]	Spain	PC	III-IV	CTx	NLR	2.455	Low	39	ND	ND
Piciucchi et al., 2017 [12]	Italy	PDAC	ND	Mixed	NLR	5	High	206	60	144
Shirai et al., 2015 [15]	Japan	PDAC	ND	ND	NLR	5	High	131	15	116
					PLR	150	Low	131	73	58
Sierzega et al., 2017 [13]	Poland	PDAC	I-III	Mixed	NLR	5	High	442	119	323
Sugiura et al., 2013 [34]	Japan	PDAC	III-IV	Mixed	NLR	4	Low	83	36	47
Sugiura et al., 2017 [9]	Japan	PDAC	III-IV	CTx	NLR	4	Low	129	62	67
Szkandera et al., 2014 [33]	Austria	PDAC	I-IV	Mixed	NLR	3.25	Low	474	247	227
Tao et al., 2016 [16]	China	PDAC	I-IV	Mixed	NLR	2.5	Low	154	84	70
					PLR	150	Low	154	81	73
Takakura et al., 2016 [26]	Japan	PC	I-III	Mixed	NLR	4.3	High	28	ND	ND
Teo et al., 2013 [40]	Ireland	PDAC	III-IV	CTx	NLR	3	Low	85	58	27
Tsujita et al., 2017 [8]	Japan	PC	II-IV	Mixed	NLR	3	Low	86	ND	ND
Vivaldi et al., 2016 [18]	Italy	PC	III-IV	CTx	NLR	4	Low	119	21	98
Wang et al., 2012 [35]	China	PDAC	I-IV	Mixed	NLR	5	High	177	32	145
Wu et al., 2016 [17]	China	PDAC	III-IV	Mixed	NLR	5	High	233	57	176
Xu et al., 2017 [38]	China	PDAC	I-IV	Mixed	NLR	3.8	Low	265	71	194
					PLR	182.1	High	265	87	178
Xue et al., 2014 [32]	Japan	PDAC	III-IV	CTx	NLR	5	High	252	40	212
					PLR	150	Low	252	148	104
Yamada et al., 2016 [20]	Japan	PC	I-IV	Mixed	NLR	3	Low	379	130	249
					PLR	150	Low	379	192	187
Yu et al., 2017 [39]	China									
Training set		PC	III-IV	CTx	NLR	3.42	Low	139	93	46
Validation set		PC	III-IV	CTx	NLR	3.42	Low	225	131	94

Tx: Treatment; PDAC: pancreatic ductal adenocarcinoma; PC: pancreatic cancer; CTx: chemotherapy; RTx: radiation therapy; NLR: neutrophil-to-lymphocyte ratio; PLR: platelet-to-lymphocyte ratio; ND: no description. “Mixed” treatment indicates surgery plus chemotherapy/radiotherapy.

**Table 2:** Estimated rates of high neutrophil-to-lymphocyte and platelet-to-lymphocyte ratios.

	Number of subset	Fixed effect (95% CI)	Heterogeneity test (*P* value)	Random effect (95% CI)	Egger's test (*P* value)
*NLR*					
Overall	29	0.422 (0.409, 0.436)	<0.001	0.379 (0.310, 0.454)	0.086
Location					
Asia	22	0.426 (0.411, 0.442)	<0.001	0.370 (0.285, 0.464)	0.067
Non-Asia	7	0.413 (0.388, 0.438)	<0.001	0.406 (0.295, 0.528)	0.855
Tumor stage					
I and II	1	0.656 (0.587, 0.720)	1.000	0.656 (0.587, 0.720)	—
III and IV	14	0.406 (0.384, 0.428)	<0.001	0.373 (0.273, 0.484)	0.375
Treatment					
Surgery	1	0.701 (0.653, 0.745)	1.000	0.701 (0.653, 0.745)	—
Chemotherapy	10	0.426 (0.401, 0.451)	<0.001	0.403 (0.276, 0.544)	0.571
Chemo- and radiotherapy	2	0.399 (0.325, 0.478)	<0.001	0.258 (0.045, 0.721)	—
Mixed	15	0.401 (0.385, 0.418)	<0.001	0.380 (0.294, 0.474)	0.359
NLR criteria					
High (>4)	14	0.246 (0.230, 0.263)	<0.001	0.237 (0.184, 0.299)	0.650
Low (≤4)	15	0.530 (0.514, 0.547)	<0.001	0.533 (0.459, 0.606)	0.932
*PLR*					
Overall	13	0.482 (0.464, 0.500)	<0.001	0.490 (0.438, 0.543)	0.523
Location					
Asia	11	0.476 (0.457, 0.495)	<0.001	0.480 (0.422, 0.539)	0.751
Non-Asia	2	0.533 (0.480, 0.586)	0.047	0.545 (0.435, 0.651)	—
Tumor stage					
III and IV	6	0.552 (0.522, 0.582)	0.028	0.542 (0.492, 0.592)	0.209
Treatment					
Chemotherapy	3	0.569 (0.530, 0.607)	0.042	0.552 (0.478, 0.624)	0.096
Chemo- and radiotherapy	2	0.555 (0.483, 0.625)	0.061	0.540 (0.400, 0.674)	—
Mixed	7	0.444 (0.422, 0.466)	<0.001	0.448 (0.387, 0.510)	0.739
PLR criteria					
High (>150)	4	0.404 (0.373, 0.435)	<0.001	0.435 (0.321, 0.556)	0.148
Low (≤150)	9	0.518 (0.497, 0.540)	0.006	0.519 (0.482, 0.557)	0.976

NLR: neutrophil-to-lymphocyte ratio; PLR: platelet-to-lymphocyte ratio; CI: confidence interval. “Mixed” treatment indicates surgery plus chemotherapy/radiotherapy.

**Table 3:** Correlation between lymphocyte-associated parameters and survival rate.

	Number of subset	Fixed effect (95% CI)	Heterogeneity test (*P* value)	Random effect (95% CI)	Egger's test (*P* value)
*NLR*					
Overall	35	1.147 (0.116, 1.180)	<0.001	1.737 (1.502, 2.009)	<0.001
Location					
Asia	26	1.129 (1.097, 1.162)	<0.001	1.763 (1.470, 2.114)	<0.001
Non-Asia	9	1.544 (1.365, 1.747)	0.054	1.626 (1.360, 1.946)	0.010
Tumor stage					
I and II	1	1.859 (1.272, 2.717)	1.000	1.859 (1.272, 2.717)	—
III and IV	17	1.733 (1.563, 1.921)	<0.001	1.929 (1.509, 2.467)	0.144
Treatment					
Surgery	1	1.510 (1.148, 1.986)	1.000	1.510 (1.148, 1.986)	—
Chemotherapy	13	1.797 (1.603, 2.015)	<0.001	2.043 (1.584, 2636)	0.061
Chemo- and radiotherapy	2	1.333 (0.917, 1.937)	0.020	0.922 (0.269, 3.162)	—
Mixed	18	1.110 (1.078, 1.143)	<0.001	1.670 (1.385, 2.013)	<0.001
NLR criteria					
High (>4)	18	1.954 (1.749, 2.183)	<0.001	2.001 (1.602, 2.499)	0.671
Low (≤4)	17	1.107 (1.075, 1.139)	<0.001	1.508 (1.285, 1.770)	<0.001
*PLR*					
Overall	14	1.009 (1.007, 1.011)	0.009	1.143 (1.037, 1.259)	0.008
Location					
Asia	11	1.009 (1.007, 1.011)	0.026	1.121 (1.010, 1.243)	0.038
Non-Asia	3	1.217 (1.013, 1.462)	0.171	1.239 (0.968, 1.587)	0.232
Tumor stage					
III and IV	6	1.155 (1.002, 1.332)	0.281	1.158 (0.983, 1.364)	0.828
Treatment					
Chemotherapy	3	1.053 (0.861, 1.287)	0.488	1.053 (0.861, 1.287)	0.203
Chemo- and radiotherapy	2	1.282 (0.937, 1.753)	0.075	1.206 (0.674, 2.158)	—
Mixed	8	1.009 (1.007, 1.011)	0.021	1.125 (1.009, 1.254)	0.043
PLR criteria					
High (>150)	5	1.009 (1.007, 1.011)	0.001	1.219 (0.992, 1.499)	0.080
Low (≤150)	9	1.105 (1.000, 1.222)	0.561	1.105 (1.000, 1.222)	0.471

NLR: neutrophil-to-lymphocyte ratio; PLR: platelet-to-lymphocyte ratio; CI: confidence interval. “Mixed” treatment indicates surgery plus chemotherapy/radiotherapy.

## Data Availability

The data supporting this meta-analysis are from previously reported studies and datasets, which have been cited. The processed data are available from the corresponding author upon request.
